# Regional Organisation and Sex‐Related Differences in Trigeminal Nerve DTI Metrics in Healthy Young Adults

**DOI:** 10.1111/ejn.70668

**Published:** 2026-09-08

**Authors:** Eduardo Rocha Arruda, Hohana G. Konell, Carlos E. Garrido Salmon

**Affiliations:** ^1^ Departamento de Neurociências e Ciências do Comportamento, Faculdade de Medicina de Ribeirão Preto Universidade de São Paulo São Paulo Brazil; ^2^ A.C. Camargo Cancer Center (CIPE) São Paulo Brazil; ^3^ Departmento de Física, Faculdade de Filosofia Ciências e Letras de Ribeirão Preto Universidade de São Paulo São Paulo Brazil; ^4^ Departamento de Imagens Médicas, Hematología e Oncología Clínica, Faculdade de Medicina de Ribeirão Preto Universidade de São Paulo São Paulo Brazil

**Keywords:** diffusion tensor imaging, fractional anisotropy, lateral asymmetry, trigeminal nerve, trigeminal neuralgia

## Abstract

Trigeminal neuralgia has been associated with microstructural alterations of the trigeminal nerve, particularly at the root entry zone, but region‐specific reference data stratified by sex in healthy individuals are lacking. This study aimed to characterise the regional organisation of the trigeminal nerve using diffusion tensor imaging (DTI) metrics and volumetry in healthy young adults, describing regional heterogeneity, lateral asymmetry and sex effects. T1‐weighted and diffusion‐weighted images from 300 Human Connectome Project participants (150 women, 150 men; 22–35 years) were used to bilaterally segment four regions of the trigeminal nerve. For each region of interest, we derived absolute and relative volumes, fractional anisotropy, mean diffusivity, axial diffusivity and radial diffusivity, computed asymmetry indices and coefficients of variation, and applied *t*‐tests, MANOVA and effect sizes to assess regional, lateral and sex‐related differences. We observed a pronounced regional gradient in fractional anisotropy, with highest values at the root entry zone and a progressive decrease towards Meckel's cave. Lateral asymmetries were generally small, and sex systematically influenced diffusion metrics and volumes in specific regions of interest, while preserving the same spatial pattern along the nerve. In healthy young adults, the trigeminal nerve exhibits a marked regional gradient in fractional anisotropy, accompanied by modest physiological lateral asymmetry and consistent sex‐related differences. These findings establish region‐ and sex‐specific reference ranges for diffusion metrics and volumes of the trigeminal nerve, providing a quantitative framework to distinguish expected variability from disease‐compatible alterations in future studies.

## Graphical abstract

DTI of 300 healthy young adults shows a marked fractional anisotropy gradient along the trigeminal nerve, peaking at the root entry zone and decreasing towards Meckel's cave. These normative, sex‐stratified metrics help distinguish physiological variability from potential microstructural abnormalities.

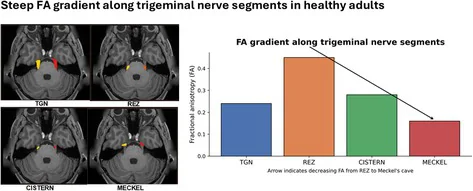

**Abbreviations**
AD: axial diffusivity
ANCOVA: analysis of covariance
CTN: classical trigeminal neuralgia
CV: coefficient of variation
DTI: diffusion tensor imaging
FA: fractional anisotropy
HCP: Human Connectome Project
ICC: intraclass correlation coefficient
ITN: idiopathic trigeminal neuralgia
MD: mean diffusivity
MRI: magnetic resonance imaging
RD: radial diffusivity
REZ: root entry zone
ROI: region of interest
SD: standard deviation
TGN: trigeminal nerve
TN: trigeminal neuralgia

## 1 Introduction

The trigeminal nerve (TGN), the largest cranial nerve, provides principal sensory innervation to the face, oral cavity and anterior head, plus motor fibres to masticatory muscles (Xie et al. 2020); trigeminal neuralgia (TN), defined in ICHD‐3 as recurrent unilateral brief electric shock‐like facial pain triggered by innocuous stimuli in one or more divisions (Headache Classification Committee of the International Headache Society (IHS) 2018), comprises classical TN (CTN; with neurovascular conflict and root entry zone [REZ] morphological alteration) and idiopathic TN (ITN; lacking compression or causal lesion despite similar phenotype; Headache Classification Committee of the International Headache Society (IHS) 2018), showing higher middle‐age female prevalence with sex factors influencing clinical and structural/radiological features (DeSouza et al. 2016; Maarbjerg et al. 2015; De Stefano et al. 2023). Microvascular compression is common in asymptomatics—insufficient alone for CTN/ITN (Maarbjerg et al. 2015)—and most imaging/surgical studies target the REZ without fully capturing heterogeneous pathway pathophysiology (DeSouza et al. 2014; DeSouza et al. 2016; Leal et al. 2011), where sex‐related morphological/physiological differences in healthy individuals may clarify underlying mechanisms for these conditions.

Diffusion tensor imaging (DTI) is a magnetic resonance technique for in vivo assessment of white matter tracts, providing metrics such as fractional anisotropy (FA), mean diffusivity (MD) and its axial (AD) and radial (RD) components, which indirectly reflect axonal organisation, myelination and processes such as demyelination and oedema (DeSouza et al. 2016; Danyluk et al. 2020; Alexander et al. 2007). In TN, DTI studies have shown, particularly at the REZ, reduced FA and increased MD, AD and RD on the affected side compared with the contralateral side and healthy controls, indicating regionally localised microstructural damage (DeSouza et al. 2016; Tohyama et al. 2021). A systematic review and subsequent studies have reinforced that most DTI investigations focus almost exclusively on the REZ and use these diffusion metrics as microstructural markers in comparisons between patients and controls (DeSouza et al. 2014; Watanabe et al. 2025; Leal et al. 2019; Zhang et al. 2018; Liu et al. 2013; DeSouza et al. 2015; Su et al. 2024; Lin et al. 2014; Wu et al. 2020; Elkholy et al. 2023).

Detailed reference data for the TGN in healthy individuals remain limited, with no prior DTI study systematically characterising FA, MD, AD, RD, absolute/relative volumes and lateral asymmetry across multiple regions in a large cohort of healthy young adults stratified by sex, leaving unclear whether TN‐reported diffusion alterations exceed healthy variability when regional differences are considered. We therefore aimed to delineate the TGN's regional organisation in healthy young adults via DTI metrics and volumetry, quantifying spatial heterogeneity of diffusion metrics/volumes across regions of interest (ROI), inter‐side asymmetry and sex effects, and establishing normative reference ranges that can inform the interpretation of diffusion changes reported in trigeminal neuralgia studies.

## 2 Methods

### 2.1 Sample and MRI Acquisition

T1w structural and DWI images were sourced from the HCP Young Adult database (Van Essen et al. 2013), selecting 300 healthy participants (22–35 years; 150 males and 150 females, balanced across age ranges 22–25, 26–30, 31–35 years) meeting HCP inclusion criteria (Van Essen et al. 2013) without selection based on TGN morphology or diffusion metrics, excluding those >35 years due to insufficient numbers for sex‐balanced groups. The HCP lacks high‐resolution CISS/T2‐SPACE sequences for neurovascular conflict detection, providing instead whole‐brain T1w, T2w and multi‐shell DWI (1.25 mm^3^ isotropic voxels, 270 directions, *b*‐values 1000/2000/3000 s/mm^2^, ~60 min acquisition) optimised for general brain imaging rather than trigeminal roots, allowing exclusion of conflicts only if causing visible anatomical alterations on conventional sequences (Van Essen et al. 2013; WU‐Minn HCP Consortium 2017). T1w acquisition used 3D MPRAGE (0.7 mm^3^ voxels, TR 2400 ms, TE 2.14 ms, TI 1000 ms, flip angle 8°; Van Essen et al. 2013), while prolonged DWI protocols maximised data quality for small regions despite limited routine clinical use (Van Essen et al. 2013).

This study analysed publicly available Human Connectome Project data and was conducted in accordance with applicable local ethics requirements; formal ethical review was considered unnecessary for this project.

### 2.2 Sampling Regions of the TGN

ROIs of the TGN were defined using classical neuroradiological landmarks that distinguish the REZ, cisternal segment and Meckel's cave (Naidich et al. 2012). In this study, standardised sampling regions rather than full segment extents were implemented to maximise anatomical specificity and reduce partial volume effects with cerebrospinal fluid.

The REZ was delineated from the emergence of the nerve at the anterolateral surface of the pons, encompassing three to four consecutive axial slices. For the cisternal sampling region, we segmented only the medial portion of the prepontine cistern, defined as two to three axial slices in which the trigeminal bundle lay clearly within the cistern, separated from both the REZ and the porus trigeminus, with stable calibre and orientation.

The Meckel region was segmented to include the TGN and ganglion within Meckel's cave, bounded posteriorly by the porus trigeminus and anteriorly by the dural walls at the petrous apex. Boundaries between the REZ, cisternal sampling region and Meckel's cave thus followed established anatomical landmarks, and restricting the cisternal ROI to its medial portion was intended to reduce contour uncertainty and increase anatomical specificity.

**FIGURE 1:**
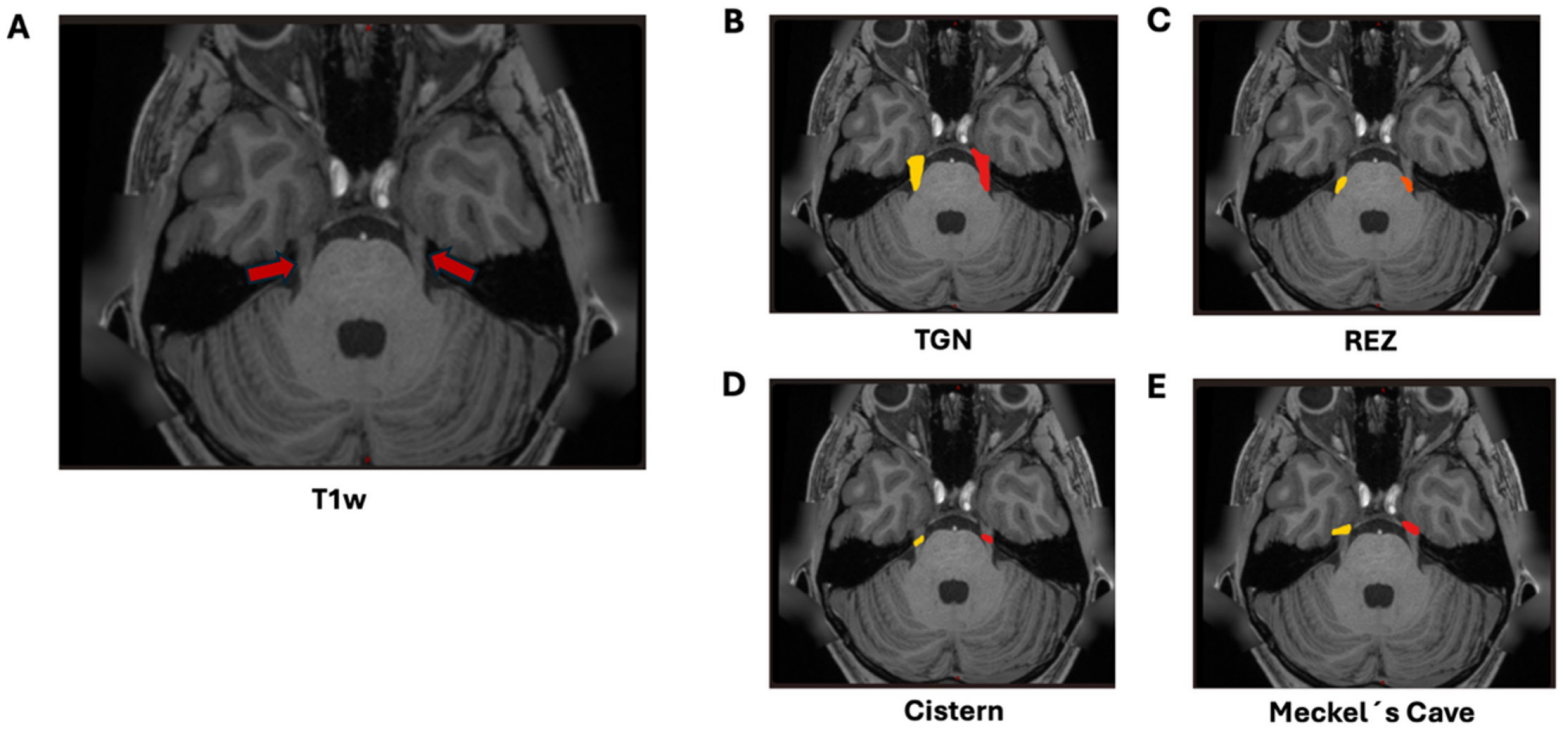
Axial view of the TGN and segmentation of the ROIs on T1w images. Axial T1w images indicating: (A) bilateral location of the TGN (red arrows); (B) bilateral segmentation of the TGN; (C) bilateral segmentation of the root; (D) bilateral segmentation of the cistern; (E) bilateral segmentation of Meckel's cave. Prepared by the authors with an image obtained from the Human Connectome Project database. ConnectomeDB. Available at: <https://db.humanconnectome.org/app/template/Index.vm>. Accessed: June 2023.

The ROIs were defined as follows: whole TGN (from the REZ to Meckel's cave), REZ, cisternal sampling region and Meckel's cave. All ROIs were visualised in MRView (MRtrix3; Tournier et al. 2019) and bilaterally segmented on axial T1w images (Figure 1A–E). The total TGN ROI comprised five to six consecutive 0.7‐mm axial slices, from the first slice in which the nerve was visible to the last, yielding a three‐dimensional thickness of approximately 3.5–4.2 mm that was similar across participants. The cisternal ROI was intentionally restricted to a medial sampling portion rather than the entire cisternal segment, to favour anatomical certainty over complete volumetric reconstruction.

Absolute ROI volume was obtained by multiplying the number of voxels by the voxel volume (0.7 × 0.7 × 0.7 mm^3^ = 0.343 mm^3^). Relative volume was calculated as ROI volume divided by each participant's intracranial volume (sum of white matter, grey matter and cerebrospinal fluid).

### 2.3 Diffusion Metrics

Diffusion images from the HCP database had undergone standard preprocessing to correct for motion, distortions and eddy‐current artefacts (Glasser et al. 2013); diffusion metric extraction in MRtrix3 (Tournier et al. 2019) involved: (i) DWI conversion to .mif format; (ii) diffusion tensor fitting within a brain mask; (iii) generation of FA, MD/ADC, AD and RD maps; (iv) T1w registration to DWI space, linear transform application to each ROI mask and thresholding in diffusion space; and (v) per‐ROI metric extraction using DWI‐space masks—defining ROIs on anatomical images independently of diffusion data and computing metrics only after registration and masking to minimise bias from prior diffusion knowledge. Intra‐ and inter‐rater reproducibility was assessed in an independent HCP subset of 40 participants (distinct from the main *n* = 300 sample), with both raters segmenting all ROIs and computing FA, MD, AD and RD before and after free‐water correction via the Pasternak et al. model (Pasternak et al. 2009) in MRtrix3 (Tournier et al. 2019); corrected metrics reproduced the uncorrected cohort's regional patterns and gradients—indicating result robustness without free‐water contamination influence—with session and rater agreement evaluated via Bland–Altman plots.

### 2.4 Asymmetry and Variability Indices

Asymmetry between right and left sides was quantified for each diffusion metric and for absolute and relative ROI volumes using an asymmetry index (A, %), computed from paired right (R)‐ and left (L)‐sided measurements within each individual:

$$A=2\times \frac{\left(R-L\right)}{\left(R+L\right)}\times 100$$

One‐sample *t*‐tests were applied to asymmetry, with interpretation focused primarily on the magnitude of asymmetry rather than *p*‐values.

Coefficients of variation (CV) were calculated for each diffusion metric to provide a relative measure of variability in the healthy cohort (Table 2).

## 3 Statistical Analysis

Statistical analyses were grouped into three families of hypotheses: regional heterogeneity among the four regions (TGN, REZ, cistern, Meckel's cave), lateral asymmetry and sex effects on diffusion metrics and volumes. Right and left measurements were treated as paired observations, and asymmetry indices were tested with one‐sample *t*‐tests against zero, with emphasis on the magnitude of asymmetry and reporting *p*‐values and 95% confidence intervals. Regional heterogeneity in FA, MD, AD and RD was assessed with Kruskal–Wallis tests followed by Dunn's tests with correction for multiple comparisons, whereas sex effects on the set of diffusion metrics and volumes were examined using MANOVA (Pillai's trace), with effect sizes and follow‐up univariate tests to characterise individual metrics.

## 4 Results

Regional and asymmetric differences were found for all diffusion and volume metrics across ROIs, indicating consistent spatial variation along the trigeminal pathway. Sex did not affect asymmetry indices, but sex‐related effects were observed for all diffusion metrics in specific ROIs, suggesting distinct patterns in males and females in particular nerve segments. Results are presented according to the main effects investigated: regional heterogeneity, lateral asymmetry and sex effects on diffusion metrics and volumes.

### 4.1 Regional Heterogeneity

**FIGURE 2:**
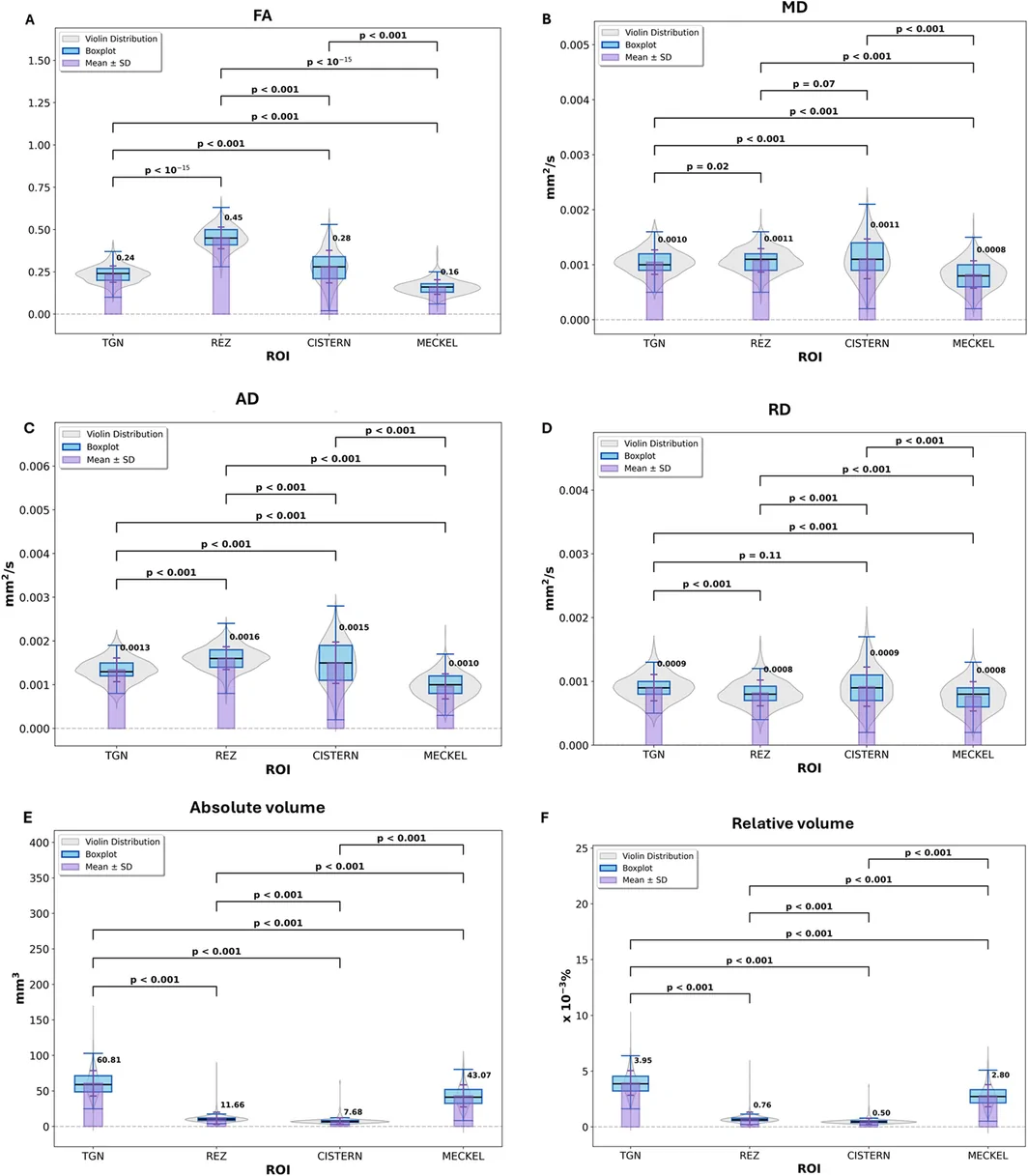
Regional differences in diffusion metrics and volume along the TGN. Distribution of FA (A), mean diffusivity (MD; B), axial diffusivity (AD; C), radial diffusivity (RD; D), absolute volume (E) and relative volume (F) in the four TGN ROIs: TGN, REZ, medial cisternal segment (CISTERN) and Meckel's cave (MECKEL). For each ROI, the grey shape depicts the density distribution (violin plot), the blue boxplot shows the median and interquartile range and the purple bar indicates the mean ± standard deviation. *p* values denote pairwise comparisons between regions, highlighting marked regional heterogeneity in both diffusion metrics and volumetric measures along the trigeminal pathway.

The REZ showed the highest FA, with lower FA in Meckel's cave and the cisternal sampling region (Table 1; Figure 2A). MD was highest in the REZ and cistern and lowest in Meckel's cave, with no significant MD difference between REZ and cistern (*p* = 0.070; Figure 2B). AD was higher in the REZ and decreased towards Meckel's cave and the cistern (*p* < 0.001; Figure 2C), whereas RD was higher in the cistern and Meckel's cave (Figure 2D). Regional variation in absolute and relative volumes was greatest in Meckel's cave, followed by the REZ and cistern (Figure 2E,F).

**TABLE 1:** DTI metrics of the TGN by ROI.

ROI	FA, mean (SD), [IC 95%]	MD, mean (SD) mm^2^/s, [IC 95%] mm^2^/s	AD, mean (SD) mm^2^/s, [IC 95%] mm^2^/s	RD, mean (SD) mm^2^/s, [IC 95%] mm^2^/s
TNG	0.24 (0.05), [0.232–0.240]	0.0010 (0.0002), [0.00103–0.00106]	0.0013 (0.0003), [0.00131–0.00136]	0.0009 (0.0002), [0.00088–0.00091]
REZ	0.45 (0.06), [0.445–0.456]	0.0011 (0.0002), [0.00106–0.00109]	0.0016 (0.0003), [0.00158–0.00162]	0.0008 (0.0002), [0.00080–0.00083]
CISTERN	0.28 (0.09), [0.273–0.288]	0.0011 (0.0004), [0.00108–0.00113]	0.0015 (0.0005), [0.00146–0.00154]	0.0009 (0.0003), [0.00089–0.00094]
MECKEL	0.16 (0.04), [0.156–0.163]	0.0008 (0.0002), [0.00080–0.00084]	0.0010 (0.0003), [0.00094–0.00098]	0.0008 (0.0002), [0.00074–0.00078]

*Note:* Mean FA and mean, axial and radial diffusivity (MD, ad, RD) values of the TGN in each ROI (TGN, REZ, CISTERN, MECKEL). Values are presented as mean (standard deviation) and 95% confidence interval.

Standardised effect sizes showed a pronounced regional gradient of DTI metrics along the TGN, with large differences in FA and axial diffusivity between ROIs and comparatively smaller changes in mean and radial diffusivity. Consistently, global ANOVA *η* ^2^ values indicated that ROI explained a large proportion of the variance in FA and AD, a moderate proportion in MD and a smaller, though systematic, proportion in RD. Detailed Cohen's *d* values for each regional pair and global *η* ^2^ for each DTI metric are presented in Supplementary Tables S1 and S2.

Table 2 reports, for each diffusion metric, the coefficient of variation (CV) as a relative measure of variability in the healthy cohort. Because CV is the ratio between standard deviation and mean expressed as a percentage, it reflects how dispersed values are around the mean and allows direct comparison of variability across FA, MD, AD and RD. Higher CVs therefore indicate greater relative variability for a given metric, whereas lower CVs indicate more stable values within the healthy sample.

**TABLE 2:** Coefficient of variation (standard deviation (SD)/mean × 100 [%]) for each diffusion metric in each region of the TGN.

	TGN	REZ	CISTERN	MECKEL
FA	20.5	14.2	34.3	27.1
MD	20.0	18.2	36.4	25.0
ad	23.1	18.8	33.3	30.0
RD	22.2	25.0	33.3	25.0

Abbreviations: AD, axial diffusivity; FA, fractional anisotropy; MD, mean diffusivity; RD, radial diffusivity.

### 4.2 Asymmetry

**FIGURE 3:**
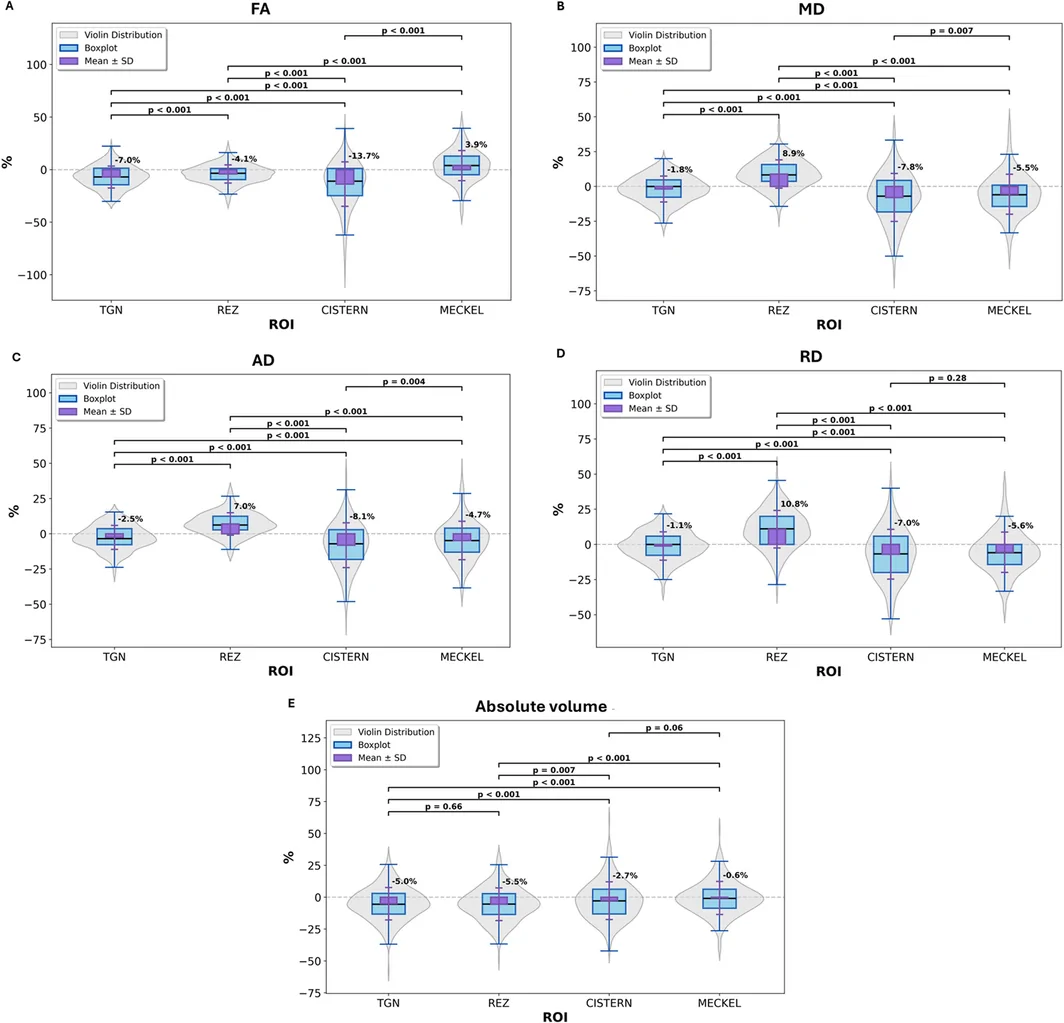
Lateral asymmetry in diffusion metrics and volume along the TGN. Percentage asymmetry differences between the right and left sides for FA (A), mean diffusivity (MD; B), axial diffusivity (AD; C), radial diffusivity (RD; D) and absolute volume (E) in the four TGN ROIs: TGN, REZ, medial cisternal segment (CISTERN) and Meckel's cave (MECKEL). Values are expressed as the percentage deviation relative to the bilateral mean of each ROI, where negative numbers indicate higher metric values on the left side and positive numbers indicate higher metric values on the right side. For each region, the grey shape represents the density distribution, the blue boxplot shows the median and interquartile range and the purple bar indicates the mean ± standard deviation of the asymmetry. *p* values denote pairwise comparisons between regions, demonstrating regional heterogeneity in asymmetry patterns of diffusion metrics and volume along the trigeminal pathway.

Asymmetry indices for FA, MD, AD, RD and absolute volume clustered around 0% in all ROIs, with means and medians nearly overlapping, indicating generally small lateral asymmetry and approximately symmetric distributions with limited influence of outliers (Figure 3A–E). For FA, the REZ and whole TGN showed slightly negative mean asymmetries, the cistern had the most negative mean asymmetry and Meckel's cave a slightly positive one, with most regional comparisons reaching statistical significance (Figure 3A). For MD, the REZ exhibited a positive mean asymmetry, whereas TGN, cistern and Meckel's cave showed negative asymmetries, again with significant differences for most regional pairs (Figure 3B). For AD, the REZ showed positive mean asymmetry and the other regions negative values (Figure 3C), and for RD, the REZ remained positively asymmetric while TGN, cistern and Meckel's cave showed negative asymmetries, with only the cistern–Meckel comparison nonsignificant (Figure 3D). For absolute volume, all ROIs exhibited slightly negative mean asymmetry, with significant differences between almost all pairs except TGN versus REZ (Figure 3E).

### 4.3 Sex Effect

**FIGURE 4:**
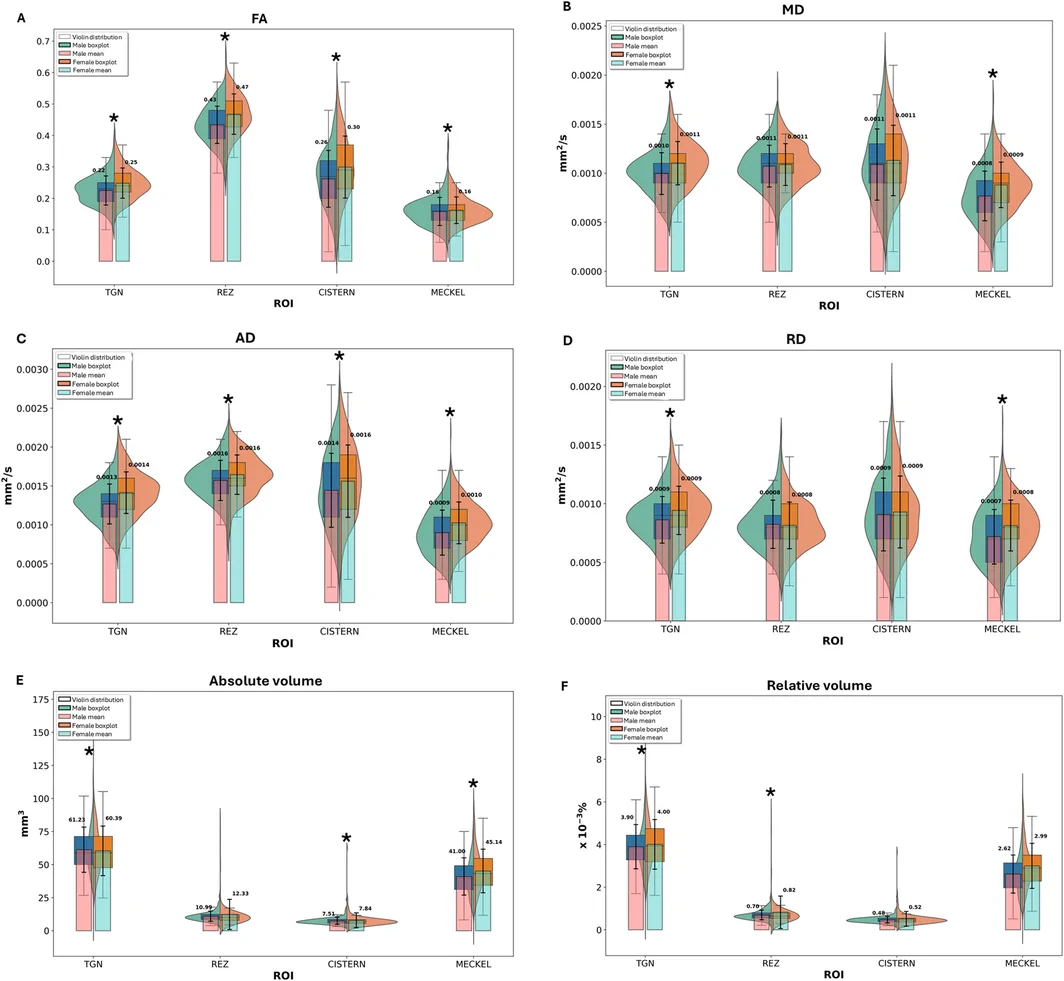
Sex differences in diffusion metrics and TGN volume. Distributions of FA (A), mean diffusivity (MD; B), axial diffusivity (ad; C), radial diffusivity (RD; D), absolute volume (E) and relative volume (F) in the four TGN ROIs: TGN, REZ, medial cisternal segment (CISTERN) and Meckel's cave (MECKEL), stratified by sex. For each ROI, green distributions represent male participants and orange distributions represent female participants; the overlaid boxplot depicts the median and interquartile range, and the coloured bars indicate the mean ± standard deviation in each group. Asterisks above the distributions denote significant differences between males and females (*p* < 0.005).

Sex had a significant multivariate effect on FA, MD, AD, RD, absolute volume and relative volume (*p* < 0.001). In Figure 4A, FA values were higher in females than males in all regions except Meckel's cave. In Figure 4B, MD was higher in females in the whole TGN and Meckel's cave (both *p* < 0.001), whereas REZ and cistern showed similar MD between sexes. In Figure 4C, AD differed by sex in all ROIs: females had higher median AD than males except in the REZ, where values were similar. In Figure 4D, RD was higher in females in Meckel's cave (*p* < 0.001), with no sex differences in TGN, REZ or cistern.

In Figure 4E, absolute volumes were larger in males in the TGN, cistern and Meckel's cave (*p* < 0.001), and in Figure 4F, relative volumes were also higher in males overall, except in the REZ, where females showed higher median relative volume. Although Figure 4A–F suggest that males and females share a similar spatial gradient of diffusion metrics along the nerve, with sex‐related shifts in magnitude in specific ROIs, the study was not designed to formally test sex × region interactions using hierarchical models; preservation of the spatial pattern between sexes should therefore be interpreted descriptively.

## 5 Discussion

Regional gradients of FA, AD, MD and RD along the TGN confirm an intrinsically heterogeneous microstructural organisation in healthy young adults, with maximal FA and AD at the REZ and progressive reductions towards Meckel's cave, whereas MD and RD change more subtly across regions; in this framework, the REZ marks the central–peripheral myelin interface, the sampled cisternal segment corresponds to fibres that are already predominantly peripheral but still highly aligned, and Meckel's cave includes the trigeminal ganglion, with lower fibre alignment and greater directional dispersion, providing a parsimonious anatomical explanation for the low FA and AD values in this region and aligning with previous descriptions of trigeminal regional architecture and DTI studies in trigeminal neuralgia (DeSouza et al. 2014; Alexander et al. 2007; Watanabe et al. 2025; DeSouza et al. 2015; Su et al. 2024).

The very large effect sizes for FA and AD between REZ, cistern and Meckel's cave indicate a robust anatomical contrast in healthy individuals and help contextualise the recurrent focus on the REZ in clinical DTI studies of trigeminal neuralgia (DeSouza et al. 2014; Alexander et al. 2007; Zhang et al. 2018; Liu et al. 2013; WU‐Minn HCP Consortium 2017), but these contrasts mainly reflect regional microstructural heterogeneity and do not support inferences about heightened vulnerability to pathology or disease predisposition, positioning our data as a quantitative reference for cautious interpretation of prior patient findings rather than direct evidence of pathophysiological mechanisms. Although RD explained a smaller share of overall variance than FA and AD, its consistently higher values in the cisternal segment and Meckel's cave than in the REZ support specific microstructural differences between segments and reinforce the complementary role of myelin‐related metrics in characterising regional gradients, matching previous trigeminal DTI reports (Watanabe et al. 2025; DeSouza et al. 2015; Su et al. 2024).

The mild, regionally modulated asymmetry pattern observed in healthy individuals, with distributions centred near 0% and small interside percentage differences, delineates the range of physiological asymmetry in this cohort and provides quantitative context for interpreting reports of more pronounced structural asymmetries in the affected nerve of patients with trigeminal neuralgia, while recognising that parametric tests may yield statistical significance even with small magnitudes, thus prioritising effect size and biological plausibility (DeSouza et al. 2014; Alexander et al. 2007; Lin et al. 2014; WU‐Minn HCP Consortium 2017).

In clinical cohorts, reductions in FA, increases in MD, AD and RD, and unilateral volumetric alterations at the REZ have been repeatedly linked to the symptomatic side and, in some studies, tracked longitudinally with responses to surgical or neuromodulatory treatments (DeSouza et al. 2014; Alexander et al. 2007; Leal et al. 2019; Lin et al. 2014; Van Essen et al. 2013); together, our reference data and these reports suggest that REZ structural asymmetry may exceed physiological levels in some patients, although this does not establish diagnostic cutoffs or validate asymmetry indices as robust disease markers on its own.

Complementarily, the asymmetry indices and CV obtained in this cohort provide a quantitative context for interpreting previous reports of more pronounced diffusion asymmetry in patients with trigeminal neuralgia, particularly at the REZ (DeSouza et al. 2014; Alexander et al. 2007; Leal et al. 2019; Zhang et al. 2018; Liu et al. 2013; DeSouza et al. 2015; Su et al. 2024; Lin et al. 2014; Wu et al. 2020; Elkholy et al. 2023; Van Essen et al. 2013). Clinical studies have described reductions in FA and increases in MD, AD and RD predominantly on the symptomatic side, sometimes accompanied by volumetric alterations and longitudinal changes following surgical or neuromodulatory interventions (DeSouza et al. 2014; Alexander et al. 2007; Leal et al. 2019; Zhang et al. 2018; Liu et al. 2013; DeSouza et al. 2015; Su et al. 2024; Lin et al. 2014; Wu et al. 2020; Elkholy et al. 2023; Van Essen et al. 2013). In this framework, the modest asymmetry pattern observed in healthy young adults suggests that more marked differences reported in clinical cohorts are likely to exceed the physiological variability expected in individuals without facial pain. Nevertheless, such an interpretation must remain cautious, as these studies involve older and heterogeneous populations and differ substantially in acquisition parameters and spatial resolution. Accordingly, the present normative data should be viewed as a quantitative reference for contextualising past and future findings rather than as diagnostic cutoffs or a definitive criterion for separating physiological variability from pathological change across the lifespan.

To further contextualise these findings, Table 3 presents the mean diffusion coefficients measured at the REZ in healthy young adults. The REZ is the most frequently studied ROI in research on TGN pathology (Leal et al. 2019; Zhang et al. 2018; Liu et al. 2013; DeSouza et al. 2015; Su et al. 2024; Lin et al. 2014; Wu et al. 2020; Elkholy et al. 2023). The diffusion coefficient values reported in the aforementioned studies were also included, allowing comparison of the present data with the expected values for healthy young adults across different diffusion metrics. Given the age differences between the historical cohorts and the present young adult sample, and the lack of lifespan normative data for TGN microstructure, this cross‐study comparison must be regarded as exploratory and illustrative rather than a definitive evaluation of pathological change.

**TABLE 3:** Mean (standard deviation, SD) values of diffusion metrics measured at the root of the TGN in healthy adults, as reported in previous papers and this study.

References	Complementary data	Mean (SD) values of diffusion metrics
Liu et al. (2013)	6 healthy controls (3 women, 3 men; aged 30– 52 years). *b* = 1000 s/mm^2^; diffusion directions = 12; field strength = 3 T; resolution = 3 × 3 × 3 mm^3^	FA = 0.51 (±0.09) MD = 1.68 (±0.25) × 10^−3^ mm^2^/s AD = 2.75 (±0.25) × 10^−3^ mm^2^/s RD = 1.11 (±0.28) × 10^−3^ mm^2^/s
Lin et al. (2014)	18 healthy controls (9 women, 9 men; mean age: 55 years). *b* = 1000 s/mm^2^; diffusion directions = 20; field strength = 3 T; resolution = 2.4 × 2.4 × 2.4 mm^3^	FA = 0.49 (±0.03) AD = 2.93 (±0.12) × 10^−3^ mm^2^/s RD = 1.08 (±0.08) × 10^−3^ mm^2^/s
Zhang et al. (2018)*	28 healthy controls (17 women, 11 men; mean age: 46 years). *b* = 1000 s/mm^2^; diffusion directions = 64; field strength = 3 T; resolution = 2 × 2 × 2 mm^3^	FA = 0.38 (±0.11) MD = 1.7 (±0.5) × 10^−3^ mm^2^/s AD = 2.4 (±0.5) × 10^−3^ mm^2^/s RD = 1.4 (±0.5) × 10^−3^ mm^2^/s
Leal et al. (2019)	6 healthy controls (3 women, 3 men; aged 22– 58 years). *b* = 1000 s/mm^2^; diffusion directions = 32; field strength = 3 T; resolution = 2 × 2 × 2 mm^3^	FA = 0.52 (±0.04) ADC = 3.84 (±0.43) × 10^−3^ mm^2^/s
Wu et al. (2020)	20 healthy controls (11 women, 9 men; mean age: 57 years). *b* = 1000 s/mm^2^; diffusion directions = 64; field strength = 3 T; resolution = 2 × 2 × 2 mm^3^	FA = 0.54 (±0.06) ADC = 3.24 (±0.43) × 10^−3^ mm^2^/s
Elkholy et al. (2023)	15 healthy controls (10 women, 5 men; aged 19–48 years). *b* = 1000 s/mm^2^; diffusion directions = 12; field strength = 1.5 T; resolution = 2 mm^3^	FA = 0.54 (±0.057)
In this study	300 subjects (150 male and 150 female; aged 22–35 years). *b* (1000, 2000 and 3000 s/mm^2^); diffusion directions = 270; field strength = 3 T; resolution = 1.25 × 1.25 × 1.25 mm^3^	FA = 0.45 (±0.06) MD = 1.1 (±0.2) × 10^−3^ mm^2^/s AD = 1.6 (±0.3) × 10^−3^ mm^2^/s RD = 0.8 (±0.2) × 10^−3^ mm^2^/s

Abbreviations: AD, axial diffusivity; ADC, apparent diffusion coefficient; *b*, *b*‐value; FA, fractional anisotropy; MD, mean diffusivity; RD, radial diffusivity.

* Mean values of diffusion metrics reported by authors.

In particular, Su et al. (2024) reported an FA gradient that decreases from the root towards the trigeminal ganglion while other diffusion metrics remain relatively uniform along the nerve, whereas the present study identified both a pronounced FA gradient and subtle along‐tract variations in MD, AD and RD. These discrepancies are unlikely to reflect sample size alone and are more plausibly driven by differences in acquisition protocols, including single‐shell *b*‐values, lower spatial resolution and fewer diffusion directions in clinical‐oriented sequences, compared with the multi‐shell, high‐resolution HCP acquisition used here.

Substantial discrepancies between diffusion metrics reported across trigeminal studies largely stem from single *b*‐value acquisitions, which overestimate diffusivity and partial volume effects from adjacent cerebrospinal fluid that reduce FA and increase ADC/MD (Danyluk et al. 2020), particularly with large voxel sizes; high spatial resolution with 1.25 mm^3^ isotropic voxels and numerous diffusion directions—as used here—are recommended to mitigate these biases, improve tensor estimation and enhance accuracy (Danyluk et al. 2020; Alexander et al. 2007).

The sex‐dependent differences observed in our metrics, with distinct FA, MD/ADC, AD, RD and volume values in specific ROIs, indicate that sex is an important determinant of normative TGN variability in healthy young adults—despite the along‐tract spatial gradient appearing similar between males and females in Figures 2 and 4, a descriptive interpretation—aligning with the higher prevalence of ITN in women and reports of sex‐related radiological and clinical features in recent studies (Maarbjerg et al. 2015; De Stefano et al. 2023), though limited here to characterising sex‐stratified reference differences in disease‐free individuals.

Our results do not demonstrate increased susceptibility to trigeminal neuralgia or distinctive radiological markers in either sex; they provide sex‐specific reference values that may inform the interpretation of studies stratifying patients by sex and emphasise the need to consider sex as a biological variable when analysing clinical and imaging findings in trigeminal neuralgia.

Volumetric differences between TGN, REZ, cistern and Meckel's cave, with larger volumes in proximal and distal segments, complement evidence of REZ structural alterations even when overall nerve morphology is preserved in trigeminal neuralgia patients with or without neurovascular conflict (DeSouza et al. 2016; DeSouza et al. 2014; Alexander et al. 2007; Elkholy et al. 2023), while the small transitional REZ with marked anisotropy and diffusivity contrast aligns with its disproportionate clinical impact and established links to neurovascular contact, DTI abnormalities and pain (Leal et al. 2011; Alexander et al. 2007; Van Essen et al. 2013; WU‐Minn HCP Consortium 2017).

In our healthy young sample, the left TGN showed larger absolute volumes across all ROIs with slightly negative mean asymmetries (particularly TGN and REZ) and near‐symmetry in Meckel's cave (Figure 3E), refining physiological volumetric asymmetry characterisation by indicating a slightly smaller right side in health—consistent with reduced affected‐side volumes in CTN with or without compression (Alexander et al. 2007; Urgošík et al. 2014)—and suggesting that pronounced clinical reductions exceed this reference range, framing a distinction between physiological asymmetry and structural remodelling in CTN or ITN even without macroscopic lesions (DeSouza et al. 2014; Su et al. 2024).

Key strengths of this work include the large healthy sample, high‐quality imaging minimising cerebrospinal fluid and susceptibility artefacts, and systematic assessment of the nerve across distinct anatomical regions.

The main limitation is manual segmentation by a single rater in the main cohort of 300 participants, rendering DTI metrics rater‐dependent and precluding formal inter‐rater reproducibility estimates; this was mitigated via complementary analysis in an independent HCP subset of 40 participants, where two raters repeated ROI segmentation before and after free‐water correction using the Pasternak et al. model (Pasternak et al. 2009) implemented in MRtrix3 (Tournier et al. 2019), yielding good Bland–Altman agreement and preservation of the main sample's regional pattern—suggesting findings are not free‐water artefacts, though not substituting formal multi‐rater reliability studies in larger samples. Additionally, histological or neuroanatomical data are lacking to validate DTI metric interpretations biologically, cisternal and Meckel's regions are CSF partial volume‐sensitive (altering absolute values), we reported conventional DTI metrics as normative references consistent with most trigeminal neuralgia studies without hierarchical mixed‐effects models or comprehensive FDR control across metrics/ROIs, recommending emphasis on consistent patterns of regional gradients, subtle physiological asymmetry and sex effects over individual *p*‐values, with findings limited to young adult normative variability without direct lifespan generalisability.

## 6 Conclusion

This study demonstrated that the TGN in healthy young adults exhibits markedly heterogeneous microstructural and volumetric organisation along its segments, with a robust gradient of FA and AD peaking at the REZ and declining towards Meckel's cave, accompanied by subtler variations in MD and RD, lateral asymmetry and segmental volumes. By establishing detailed sex‐ and region‐stratified reference ranges for FA, MD, AD, RD and volumes—revealing small, homogeneously distributed physiological asymmetries—it provides a robust quantitative framework to differentiate expected variation from potentially pathological patterns, enabling critical interpretation of prior and future trigeminal microstructure investigations in clinical and experimental settings.

Furthermore, it underscores sex‐related differences in trigeminal neuroimaging, as sex‐stratified analyses uncovered systematic diffusion and volume variations that pooled analyses may obscure, advocating sex‐specific normative values from healthy samples to minimise bias and enhance precision in future studies.

## Author Contributions

**Eduardo Rocha Arruda:** conceptualization, investigation, funding acquisition, writing – original draft, methodology, validation, visualization, writing – review and editing, formal analysis, data curation, software. **Hohana G. Konell:** conceptualization, investigation, methodology, validation, visualization, writing – review and editing, software, formal analysis, data curation. **Carlos E. Garrido Salmon:** conceptualization, investigation, methodology, validation, visualization, writing – review and editing, software, formal analysis, supervision, data curation.

## Funding

Eduardo Rocha Arruda received financial support from Coordenação de Aperfeiçoamento de Pessoal de Nível Superior (CAPES; Finance Code 88887.822239/2023‐00). Hohana G. Konell received financial support from Fundação de Apoio ao Ensino, Pesquisa e Assistência do Hospital das Clínicas da Faculdade de Medicina de Ribeirão Preto da Universidade de São Paulo (FAEPA; No. 243/25). Carlos E. Garrido Salmon received financial support from the Conselho Nacional de Desenvolvimento Científico e Tecnológico (CNPq; Project 310,618/2021‐5).

## Ethics Statement

The study was exempted from ethical review by the CEP/CONEP system.

## Consent

The authors have nothing to report.

## Conflicts of Interest

The authors declare no conflicts of interest.

## Supporting information


**Appendix S1:** Mean, median and standard deviation of the raw values of diffusion metrics measured bilaterally in each region of interest of the trigeminal nerve. Mean, median and standard deviation of the raw values of fractional anisotropy (FA) measured bilaterally in each region of interest of the trigeminal nerve. Abbreviations: L, left side; R, right side; REZ, root entry zone; TGN, trigeminal nerve.

**Table S1:** Effect sizes (Cohen's *d*) between regions of interest for DTI metrics.
**Table S2:** Global ANOVA effect sizes (η2) for each DTI metric.

## Data Availability

The data supporting the findings of this study are not publicly available but are available from the corresponding author upon reasonable request.
