# Supplementary material for: Regional Organisation and Sex‐Related Differences in Trigeminal Nerve DTI Metrics in Healthy Young Adults
Source: Eur J Neurosci. 2026 Sep 8;64(5):e70668. doi: 10.1111/ejn.70668 (PMC13551375; doi:10.1111/ejn.70668)
Supplement: Supplementary file 1 [file EJN-64-0-s001.docx]

Appenddix A - Mean, median and standard deviation of the raw values of diffusion metrics measured bilaterally in each region of interest of the trigeminal nerve. Mean, median and standard deviation of the raw values of fractional anisotropy (FA) measured bilaterally in each region of interest of the trigeminal nerve. TGN – trigeminal nerve; REZ – root entry zone; R – right side; L – left side.

| Region | Age | Sex | N | Mean | Median | Standard deviation |
| --- | --- | --- | --- | --- | --- | --- |
| TGN-R | 22-25 | Men | 50 | 0,2036 | 0,2000 | 0,0459 |
|  |  | Women | 50 | 0,2386 | 0,2300 | 0,0448 |
|  | 26-30 | Men | 50 | 0,2060 | 0,2000 | 0,0390 |
|  |  | Women | 50 | 0,2350 | 0,2400 | 0,0387 |
|  | 31-35 | Men | 50 | 0,2124 | 0,2100 | 0,0440 |
|  |  | Women | 50 | 0,2288 | 0,2200 | 0,0550 |
| TGN-L | 22-25 | Men | 50 | 0,2440 | 0,2500 | 0,0469 |
|  |  | Women | 50 | 0,2648 | 0,2550 | 0,0478 |
|  | 26-30 | Men | 50 | 0,2468 | 0,2500 | 0,0438 |
|  |  | Women | 50 | 0,2646 | 0,2600 | 0,0424 |
|  | 31-35 | Men | 50 | 0,2370 | 0,2400 | 0,0371 |
|  |  | Women | 50 | 0,2586 | 0,2500 | 0,0470 |
| REZ-R | 22-25 | Men | 50 | 0,4182 | 0,4150 | 0,0586 |
|  |  | Women | 50 | 0,4542 | 0,4500 | 0,0638 |
|  | 26-30 | Men | 50 | 0,4132 | 0,4050 | 0,0549 |
|  |  | Women | 50 | 0,4448 | 0,4400 | 0,0637 |
|  | 31-35 | Men | 50 | 0,4093 | 0,4200 | 0,0571 |
|  |  | Women | 50 | 0,4586 | 0,4600 | 0,0692 |
| REZ-L | 22-25 | Men | 50 | 0,4566 | 0,4550 | 0,0591 |
|  |  | Women | 50 | 0,4816 | 0,4750 | 0,0589 |
|  | 26-30 | Men | 50 | 0,4560 | 0,4550 | 0,0530 |
|  |  | Women | 50 | 0,4876 | 0,4850 | 0,0577 |
|  | 31-35 | Men | 50 | 0,4506 | 0,4500 | 0,0525 |
|  |  | Women | 50 | 0,4806 | 0,4900 | 0,0637 |
| Cistern-R | 22-25 | Men | 50 | 0,2114 | 0,2150 | 0,0942 |
|  |  | Women | 50 | 0,2638 | 0,2700 | 0,0954 |
|  | 26-30 | Men | 50 | 0,2302 | 0,2250 | 0,0786 |
|  |  | Women | 50 | 0,2684 | 0,2700 | 0,0761 |
|  | 31-35 | Men | 50 | 0,2842 | 0,2850 | 0,0743 |
|  |  | Women | 50 | 0,2648 | 0,2700 | 0,1064 |
| Cistern-L | 22-25 | Men | 50 | 0,2990 | 0,3000 | 0,0705 |
|  |  | Women | 50 | 0,3440 | 0,3450 | 0,0993 |
|  | 26-30 | Men | 50 | 0,9994 | 0,2900 | 4,9073 |
|  |  | Women | 50 | 0,3236 | 0,3250 | 0,0864 |
|  | 31-35 | Men | 50 | 0,8318 | 0,2250 | 4,3551 |
|  |  | Women | 50 | 0,3328 | 0,3200 | 0,0925 |
| M.Cave-R | 22-25 | Men | 50 | 0,1564 | 0,1600 | 0,0457 |
|  |  | Women | 50 | 0,1732 | 0,1700 | 0,0470 |
|  | 26-30 | Men | 50 | 0,1696 | 0,1800 | 0,0472 |
|  |  | Women | 50 | 0,1790 | 0,1700 | 0,0501 |
|  | 31-35 | Men | 50 | 0,1586 | 0,1600 | 0,0369 |
|  |  | Women | 50 | 0,1630 | 0,1600 | 0,0400 |
| M.Cave-L | 22-25 | Men | 50 | 0,1564 | 0,1500 | 0,0530 |
|  |  | Women | 50 | 0,1512 | 0,1500 | 0,0350 |
|  | 26-30 | Men | 50 | 0,1612 | 0,1600 | 0,0468 |
|  |  | Women | 50 | 0,1560 | 0,1500 | 0,0356 |
|  | 31-35 | Men | 50 | 0,1474 | 0,1500 | 0,0349 |
|  |  | Women | 50 | 0,1512 | 0,1500 | 0,0376 |
|  |  |  |  |  |  |  |
| Region | Age | Sex | N | Mean | Median | Standard deviation |
| TGN-R | 22-25 | Men | 50 | 0,0010 | 0,0010 | 0,0002 |
|  |  | Women | 50 | 0,0010 | 0,0010 | 0,0002 |
|  | 26-30 | Men | 50 | 0,0010 | 0,0010 | 0,0002 |
|  |  | Women | 50 | 0,0012 | 0,0012 | 0,0002 |
|  | 31-35 | Men | 50 | 0,0010 | 0,0010 | 0,0002 |
|  |  | Women | 50 | 0,0011 | 0,0011 | 0,0002 |
| TGN-L | 22-25 | Men | 50 | 0,0010 | 0,0010 | 0,0002 |
|  |  | Women | 50 | 0,0011 | 0,0011 | 0,0002 |
|  | 26-30 | Men | 50 | 0,0010 | 0,0010 | 0,0002 |
|  |  | Women | 50 | 0,0011 | 0,0011 | 0,0002 |
|  | 31-35 | Men | 50 | 0,0010 | 0,0010 | 0,0002 |
|  |  | Women | 50 | 0,0011 | 0,0011 | 0,0002 |
| REZ-R | 22-25 | Men | 50 | 0,0012 | 0,0011 | 0,0002 |
|  |  | Women | 50 | 0,0012 | 0,0011 | 0,0002 |
|  | 26-30 | Men | 50 | 0,0012 | 0,0012 | 0,0002 |
|  |  | Women | 50 | 0,0012 | 0,0012 | 0,0002 |
|  | 31-35 | Men | 50 | 0,0012 | 0,0012 | 0,0002 |
|  |  | Women | 50 | 0,0012 | 0,0012 | 0,0002 |
| REZ-L | 22-25 | Men | 50 | 0,0010 | 0,0010 | 0,0001 |
|  |  | Women | 50 | 0,0010 | 0,0010 | 0,0002 |
|  | 26-30 | Men | 50 | 0,0010 | 0,0009 | 0,0002 |
|  |  | Women | 50 | 0,0010 | 0,0009 | 0,0001 |
|  | 31-35 | Men | 50 | 0,0010 | 0,0010 | 0,0002 |
|  |  | Women | 50 | 0,0010 | 0,0010 | 0,0002 |
| Cistern-R | 22-25 | Men | 50 | 0,0010 | 0,0010 | 0,0003 |
|  |  | Women | 50 | 0,0009 | 0,0009 | 0,0003 |
|  | 26-30 | Men | 50 | 0,0010 | 0,0009 | 0,0004 |
|  |  | Women | 50 | 0,0011 | 0,0011 | 0,0003 |
|  | 31-35 | Men | 50 | 0,0010 | 0,0010 | 0,0003 |
|  |  | Women | 50 | 0,0011 | 0,0011 | 0,0004 |
| Cistern-L | 22-25 | Men | 50 | 0,0012 | 0,0012 | 0,0003 |
|  |  | Women | 50 | 0,0012 | 0,0012 | 0,0003 |
|  | 26-30 | Men | 50 | 0,0012 | 0,0011 | 0,0003 |
|  |  | Women | 50 | 0,0012 | 0,0012 | 0,0003 |
|  | 31-35 | Men | 50 | 0,0012 | 0,0011 | 0,0004 |
|  |  | Women | 50 | 0,0012 | 0,0012 | 0,0004 |
| M.Cave-R | 22-25 | Men | 50 | 0,0007 | 0,0007 | 0,0003 |
|  |  | Women | 50 | 0,0008 | 0,0008 | 0,0002 |
|  | 26-30 | Men | 50 | 0,0007 | 0,0007 | 0,0002 |
|  |  | Women | 50 | 0,0009 | 0,0009 | 0,0003 |
|  | 31-35 | Men | 50 | 0,0008 | 0,0008 | 0,0002 |
|  |  | Women | 50 | 0,0008 | 0,0008 | 0,0002 |
| M.Cave-L | 22-25 | Men | 50 | 0,0008 | 0,0008 | 0,0003 |
|  |  | Women | 50 | 0,0009 | 0,0009 | 0,0002 |
|  | 26-30 | Men | 50 | 0,0008 | 0,0008 | 0,0003 |
|  |  | Women | 50 | 0,0010 | 0,0010 | 0,0002 |
|  | 31-35 | Men | 50 | 0,0008 | 0,0008 | 0,0003 |
|  |  | Women | 50 | 0,0009 | 0,0009 | 0,0002 |
|  |  |  |  |  |  |  |
| Region | Age | Sex | N | Mean | Median | Standard deviation |
| TGN-R | 22-25 | Men | 50 | 0,0012 | 0,0012 | 0,0002 |
|  |  | Women | 50 | 0,0013 | 0,0013 | 0,0002 |
|  | 26-30 | Men | 50 | 0,0012 | 0,0013 | 0,0003 |
|  |  | Women | 50 | 0,0015 | 0,0015 | 0,0003 |
|  | 31-35 | Men | 50 | 0,0012 | 0,0012 | 0,0002 |
|  |  | Women | 50 | 0,0014 | 0,0014 | 0,0003 |
| TGN-L | 22-25 | Men | 50 | 0,0013 | 0,0013 | 0,0002 |
|  |  | Women | 50 | 0,0014 | 0,0014 | 0,0003 |
|  | 26-30 | Men | 50 | 0,0013 | 0,0013 | 0,0003 |
|  |  | Women | 50 | 0,0015 | 0,0015 | 0,0002 |
|  | 31-35 | Men | 50 | 0,0013 | 0,0013 | 0,0003 |
|  |  | Women | 50 | 0,0017 | 0,0017 | 0,0002 |
| REZ-R | 22-25 | Men | 50 | 0,0018 | 0,0017 | 0,0003 |
|  |  | Women | 50 | 0,0017 | 0,0017 | 0,0003 |
|  | 26-30 | Men | 50 | 0,0017 | 0,0017 | 0,0003 |
|  |  | Women | 50 | 0,0017 | 0,0017 | 0,0002 |
|  | 31-35 | Men | 50 | 0,0017 | 0,0017 | 0,0002 |
|  |  | Women | 50 | 0,0018 | 0,0018 | 0,0002 |
| REZ-L | 22-25 | Men | 50 | 0,0015 | 0,0015 | 0,0002 |
|  |  | Women | 50 | 0,0016 | 0,0016 | 0,0002 |
|  | 26-30 | Men | 50 | 0,0014 | 0,0015 | 0,0002 |
|  |  | Women | 50 | 0,0015 | 0,0015 | 0,0002 |
|  | 31-35 | Men | 50 | 0,0014 | 0,0015 | 0,0002 |
|  |  | Women | 50 | 0,0015 | 0,0016 | 0,0002 |
| Cistern-R | 22-25 | Men | 50 | 0,0013 | 0,0013 | 0,0004 |
|  |  | Women | 50 | 0,0013 | 0,0012 | 0,0004 |
|  | 26-30 | Men | 50 | 0,0013 | 0,0013 | 0,0005 |
|  |  | Women | 50 | 0,0015 | 0,0015 | 0,0004 |
|  | 31-35 | Men | 50 | 0,0013 | 0,0013 | 0,0004 |
|  |  | Women | 50 | 0,0016 | 0,0015 | 0,0005 |
| Cistern-L | 22-25 | Men | 50 | 0,0016 | 0,0016 | 0,0005 |
|  |  | Women | 50 | 0,0017 | 0,0017 | 0,0004 |
|  | 26-30 | Men | 50 | 0,0016 | 0,0016 | 0,0005 |
|  |  | Women | 50 | 0,0017 | 0,0016 | 0,0004 |
|  | 31-35 | Men | 50 | 0,0015 | 0,0014 | 0,0006 |
|  |  | Women | 50 | 0,0017 | 0,0017 | 0,0005 |
| M.Cave-R | 22-25 | Men | 50 | 0,0008 | 0,0008 | 0,0003 |
|  |  | Women | 50 | 0,0009 | 0,0009 | 0,0003 |
|  | 26-30 | Men | 50 | 0,0008 | 0,0008 | 0,0003 |
|  |  | Women | 50 | 0,0010 | 0,0010 | 0,0003 |
|  | 31-35 | Men | 50 | 0,0009 | 0,0009 | 0,0003 |
|  |  | Women | 50 | 0,0009 | 0,0009 | 0,0002 |
| M.Cave-L | 22-25 | Men | 50 | 0,0009 | 0,0009 | 0,0003 |
|  |  | Women | 50 | 0,0010 | 0,0010 | 0,0002 |
|  | 26-30 | Men | 50 | 0,0010 | 0,0010 | 0,0003 |
|  |  | Women | 50 | 0,0011 | 0,0011 | 0,0003 |
|  | 31-35 | Men | 50 | 0,0009 | 0,0009 | 0,0003 |
|  |  | Women | 50 | 0,0011 | 0,0011 | 0,0003 |
| Region | Age | Sex | N | Mean | Median | Standard deviation |
| TGN-R | 22-25 | Men | 50 | 0,0008 | 0,0009 | 0,0002 |
|  |  | Women | 50 | 0,0009 | 0,0009 | 0,0002 |
|  | 26-30 | Men | 50 | 0,0009 | 0,0009 | 0,0002 |
|  |  | Women | 50 | 0,0010 | 0,0010 | 0,0002 |
|  | 31-35 | Men | 50 | 0,0008 | 0,0009 | 0,0002 |
|  |  | Women | 50 | 0,0009 | 0,0010 | 0,0002 |
| TGN-L | 22-25 | Men | 50 | 0,0009 | 0,0009 | 0,0002 |
|  |  | Women | 50 | 0,0009 | 0,0009 | 0,0002 |
|  | 26-30 | Men | 50 | 0,0009 | 0,0009 | 0,0002 |
|  |  | Women | 50 | 0,0010 | 0,0009 | 0,0002 |
|  | 31-35 | Men | 50 | 0,0009 | 0,0009 | 0,0002 |
|  |  | Women | 50 | 0,0009 | 0,0009 | 0,0002 |
| REZ-R | 22-25 | Men | 50 | 0,0009 | 0,0009 | 0,0002 |
|  |  | Women | 50 | 0,0009 | 0,0009 | 0,0002 |
|  | 26-30 | Men | 50 | 0,0010 | 0,0010 | 0,0002 |
|  |  | Women | 50 | 0,0009 | 0,0009 | 0,0002 |
|  | 31-35 | Men | 50 | 0,0009 | 0,0009 | 0,0002 |
|  |  | Women | 50 | 0,0009 | 0,0009 | 0,0002 |
| REZ-L | 22-25 | Men | 50 | 0,0007 | 0,0007 | 0,0001 |
|  |  | Women | 50 | 0,0008 | 0,0008 | 0,0002 |
|  | 26-30 | Men | 50 | 0,0007 | 0,0007 | 0,0002 |
|  |  | Women | 50 | 0,0007 | 0,0007 | 0,0001 |
|  | 31-35 | Men | 50 | 0,0007 | 0,0007 | 0,0001 |
|  |  | Women | 50 | 0,0007 | 0,0007 | 0,0002 |
| Cistern-R | 22-25 | Men | 50 | 0,0008 | 0,0009 | 0,0002 |
|  |  | Women | 50 | 0,0007 | 0,0007 | 0,0003 |
|  | 26-30 | Men | 50 | 0,0008 | 0,0008 | 0,0003 |
|  |  | Women | 50 | 0,0010 | 0,0010 | 0,0003 |
|  | 31-35 | Men | 50 | 0,0008 | 0,0008 | 0,0003 |
|  |  | Women | 50 | 0,0010 | 0,0009 | 0,0003 |
| Cistern-L | 22-25 | Men | 50 | 0,0010 | 0,0011 | 0,0003 |
|  |  | Women | 50 | 0,0009 | 0,0010 | 0,0003 |
|  | 26-30 | Men | 50 | 0,0009 | 0,0009 | 0,0003 |
|  |  | Women | 50 | 0,0010 | 0,0010 | 0,0003 |
|  | 31-35 | Men | 50 | 0,0010 | 0,0009 | 0,0004 |
|  |  | Women | 50 | 0,0010 | 0,0010 | 0,0004 |
| M.Cave-R | 22-25 | Men | 50 | 0,0007 | 0,0006 | 0,0002 |
|  |  | Women | 50 | 0,0007 | 0,0007 | 0,0002 |
|  | 26-30 | Men | 50 | 0,0007 | 0,0006 | 0,0002 |
|  |  | Women | 50 | 0,0008 | 0,0008 | 0,0002 |
|  | 31-35 | Men | 50 | 0,0007 | 0,0008 | 0,0002 |
|  |  | Women | 50 | 0,0007 | 0,0007 | 0,0002 |
| M.Cave-L | 22-25 | Men | 50 | 0,0007 | 0,0007 | 0,0002 |
|  |  | Women | 50 | 0,0008 | 0,0008 | 0,0002 |
|  | 26-30 | Men | 50 | 0,0008 | 0,0008 | 0,0002 |
|  |  | Women | 50 | 0,0009 | 0,0009 | 0,0002 |
|  | 31-35 | Men | 50 | 0,0007 | 0,0007 | 0,0003 |
|  |  | Women | 50 | 0,0009 | 0,0009 | 0,0002 |
