# Supplementary material for: Regional Organisation and Sex‐Related Differences in Trigeminal Nerve DTI Metrics in Healthy Young Adults
Source: Eur J Neurosci. 2026 Sep 8;64(5):e70668. doi: 10.1111/ejn.70668 (PMC13551375; doi:10.1111/ejn.70668)
Supplement: Supplementary file 2 [file EJN-64-0-s002.docx]

**Table 1. Effect sizes (Cohen’s d) between regions of interest for DTI metrics.**

| **Diffusion metric** | **Comprison between ROIs** | **Cohen´s d** | **Interpretation** |
| --- | --- | --- | --- |
| FA | TGN vs REZ | −3,77 | Very large effect size |
| FA | REZ vs cisterna | 2,08 | Very large effect size |
| FA | Cisterna vs Meckel | 1,61 | Large effect size |
| MD | TGN vs REZ | −0,14 | Small effect size |
| MD | REZ vs cisterna | −0,10 | Small effect size |
| MD | Cisterna vs Meckel | 0,92 | Large effect size |
| AD | TGN vs REZ | −1,01 | Large effect size |
| AD | REZ vs cisterna | 0,28 | Small to moderate effect size |
| AD | Cisterna vs Meckel | 1,38 | Very large effect size |
| RD | TGN vs REZ | 0,41 | Small to moderate effect size |
| RD | REZ vs cisterna | −0,38 | Small to moderate effect size |
| RD | Cisterna vs Meckel | 0,56 | Moderate effect size |

Pairwise Cohen’s d values comparing fractional anisotropy (FA), mean diffusivity (MD), axial diffusivity (AD), and radial diffusivity (RD) between trigeminal nerve regions of interest (TGN, REZ, CISTERN, MECKEL), together with qualitative interpretation of effect size magnitude (small, small to moderate, moderate, large, very large).

**Table 2. Global ANOVA effect sizes (η²) for each DTI metric.**

| **Diffusion metric** | **η²** | **Interpretation** |
| --- | --- | --- |
| FA | 0,7207 | Very large: ROI accounts for a high proportion of the variance |
| MD | 0,1499 | Moderate: regional effect on mean diffusivity |
| AD | 0,3501 | Large: marked axial gradient between ROIs |
| RD | 0,0630 | Small to moderate: more subtle radial differences |

Global η² values from one-way ANOVAs assessing the contribution of region of interest (ROI) to the variance in fractional anisotropy (FA), mean diffusivity (MD), axial diffusivity (AD), and radial diffusivity (RD), accompanied by qualitative interpretation of effect size magnitude (very large, moderate, large, small to moderate).
